# Current Trends in the Surgical Management of Yolk Sac Tumors

**DOI:** 10.3390/curroncol31110510

**Published:** 2024-11-06

**Authors:** Małgorzata Edyta Wojtyś, Konstantinos Kostopanagiotou, Dawid Kordykiewicz, Patryk Skórka, Alexandros Polykarpos Gkioulekas, Julia Augusta Guimarães Dourado, Janusz Wójcik, Periklis Tomos

**Affiliations:** 1 Department of Thoracic Surgery and Transplantation, Pomeranian Medical University in Szczecin, Alfreda Sokołowskiego 11, 70-891 Szczecin, Poland; 2 Department of Thoracic Surgery, ‘Attikon’ University Hospital of Athens, 12462 Athens, Greece; 3 Faculty of Medicine, Universitatea Ovidius Constanţa, Bulevardul Mamaia 124, 900527 Constanța, Romania

**Keywords:** yolk sac tumor, endodermal sinus tumor, non-seminomatous germ cell tumor, mediastinal neoplasms

## Abstract

Mediastinal yolk sac tumors (YST) constitute a highly malignant subtype of primary non-seminomatous germ cell tumors, which are often locally advanced and unresectable at diagnosis. Due to their rarity and aggressiveness, there is not yet a standard optimal treatment approach. A widely employed multimodal strategy involves neoadjuvant cisplatin-based combination chemotherapy followed by consolidation surgery. Tumor infiltration into the lungs and adjacent cardiovascular structures is common, such that surgical intervention typically requires extensive resections, performed either by sternotomy or thoracotomy. For this review, we extensively searched the PubMed and Embase medical databases, identifying articles describing surgical treatment of mediastinal yolk sac tumors. The current literature provides limited details regarding the utilized surgical procedures, leaving clinicians without comprehensive guidance about the demanding nature of these resections. Here, we present a narrative description of the range of current surgical procedures. By highlighting these techniques, we provide a comprehensive overview of the current surgical landscape, thereby helping clinicians understand the potential complexities and considerations involved in managing these rare and aggressive tumors.

## 1. Introduction

Germ cell tumors (GCTs) comprise 4–10% of all mediastinal tumors among adults, and up to 25% among children [1,2]. These malignancies in the mediastinum are histologically classified following the same schema used for gonadal GCTs: seminomatous tumors and nonseminomatous tumors, including yolk sac tumors (YSTs) and teratomas [3]. YSTs (also termed endodermal sinus tumors) are germ-cell-derived neoplasms. Despite their gonadal origin, 5% of germ cell tumors arise in extragonadal locations, predominantly in the mediastinum (50–70% of such cases) [4,5]. These extragonadal tumors constitute approximately 1% of all mediastinal neoplasms and are most frequently located in the anterior mediastinum [6,7]. They are thought to arise from abnormal cell migration during embryogenesis and were first described in 1967 by Teilmann et al. [8]. These tumors are most often incidentally detected or diagnosed after a period of atypical symptoms and follow a highly aggressive course, involving rapid and extensive local expansion and infiltration, and early metastases in the lungs, liver, brain, or bones [9].

The affected patients are primarily young males (mean age of 28 years), and commonly present with chest pain and dyspnea [6,10,11]. However, cases in elderly patients have also been reported [1,12]. Clinical symptoms are nonspecific—potentially including weight loss, fever, fatigue, or dysphagia, and can be associated with a variety of conditions. Due to their characteristically late diagnosis, YSTs are often detected after they have reached a substantial size, typically exceeding 12 cm in diameter [13]. Tumors of this size can cause compression to the surrounding structures, potentially leading to complications such as superior vena cava syndrome.

Yolk sac tumors exhibit variable histopathologic presentation. The most characteristic reticular type features multiple communicating channels, perivascular mantles containing Schiller–Duval bodies, and intra- and extracellular hyaline globules, all arranged within a loose reticular network [14]. In their review, Moran et al. also described different patterns, including solid areas with marked nuclear pleomorphism, prominent spindling of the cells, an “intestinal pattern” characterized by villous-like projections lined with tumor cells, and a “hepatoid pattern” resembling liver tissue [13].

The current literature, including published series and case reports, largely lacks specific detailed information regarding surgical management, leaving clinicians without comprehensive guidance about the complexity of these resections. In contemporary treatment, surgery is generally an important adjunct to cisplatin-based chemotherapy. It is not uncommon to perceive YST resections as typical mediastinal mass resections with typical risk and complexity; however, the situation is different. Our goal in this review is to present a narrative description of the range of available surgical options. This comprehensive approach will assist clinicians in understanding the potential perioperative and operative complexities and considerations.

## 2. Materials and Methods

### 2.1. The Sources and Search Strategy

For this review, we extensively searched the PubMed and Embase databases up until August 2024. The subject researched was “mediastinal yolk sac tumor”, along with the following terms: endodermal sinus tumor, surgery, cisplatin, and chemotherapy. The research was conducted according to eligibility criteria defined using the PICOTS framework. Table 1 presents the inclusion criteria.

### 2.2. Selection and Data Collection Process

**Figure 1:**
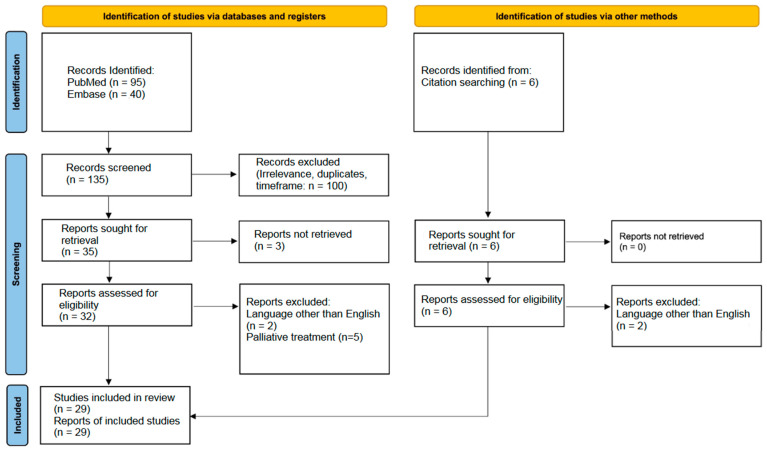
Prisma flowchart.

Following the above-described research strategy, we screened the primary results from PubMed and Embase databases. The authors examined the primary articles and conducted a full-text evaluation, excluding articles that did not meet the eligibility criteria. The extracted data included study-specific information (year of publication, country of authors), followed by individual patients’ data (e.g., symptoms, diagnosis, treatment, and follow-up). The Prisma flowchart demonstrates the literature search strategy (Figure 1).

## 3. Diagnostic Imaging and Biomarkers

**Figure 2:**
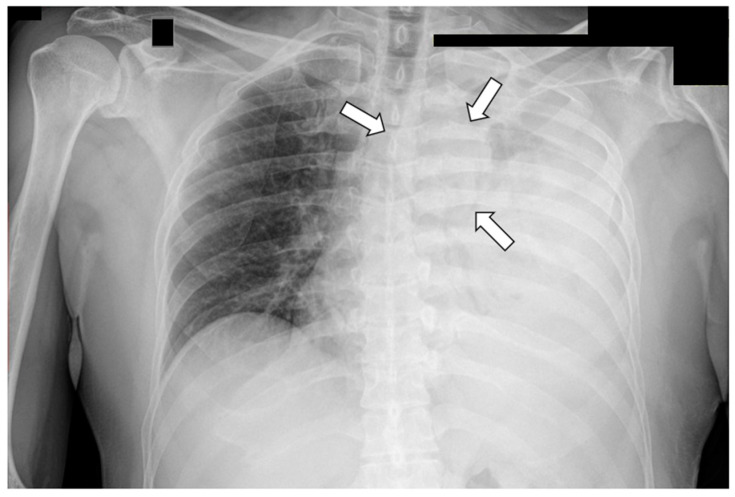
X-ray image; Initial diagnosis of parapneumonic effusion in a previously healthy middle-aged male with chest discomfort and limiting dyspnoea. A careful inspection of the CXR identifies a solid paratracheal area raising concerns of neoplasia (white arrows).

YST diagnosis relies on a combination of clinical symptoms, physical examination, imaging studies, and laboratory blood tests. With larger masses, radiological imaging may reveal a lobulated poorly marginated mediastinal mass with heterogeneous consistency. Traditionally, detection was based on chest X-rays (Figure 2), and mediastinal masses could also frequently be identified by echocardiography.

**Figure 3:**
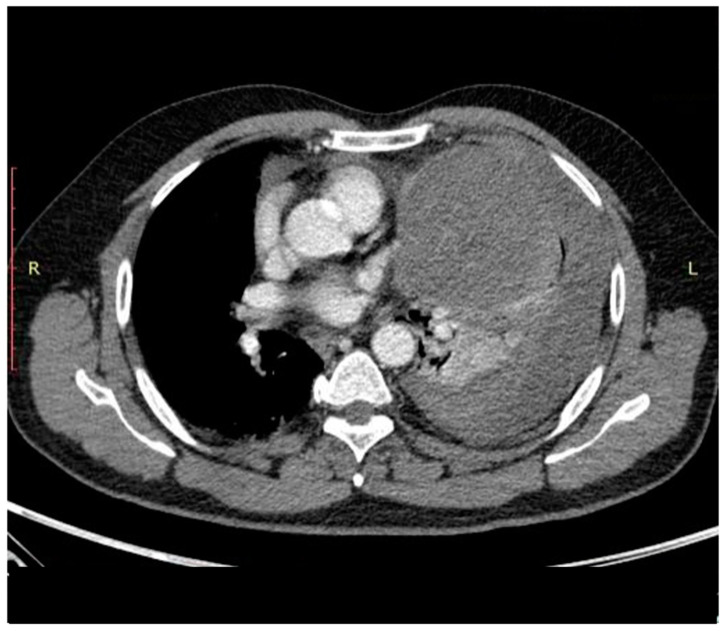
CT axial plane; Computed tomography identifies a soft-tissue mass of the left hemithorax compressing the left lung with non-specific diagnostic clues. Mediastinal involvement is best assessed by MRI.

**Figure 4:**
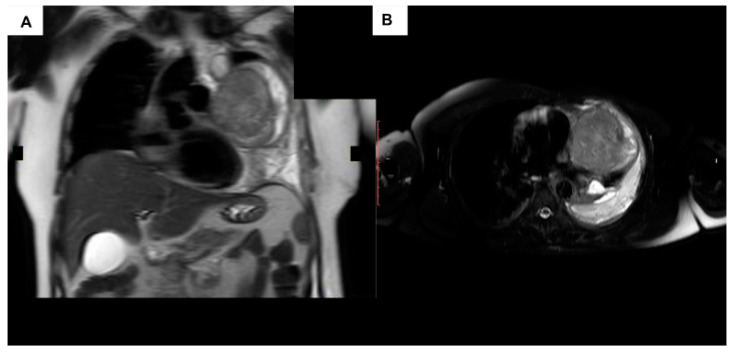
(**A**) MRI coronal plane; (**B**) MRI axial plane; Magnetic resonance images for the mass do not recognize infiltration of the great vessels; however, if the establishment of cardiopulmonary by-pass is anticipated, there will be limited exposed area for cannulation.

However, at this time, definitive diagnosis relies on computed tomography (CT) (Figure 3) and subsequent magnetic resonance imaging (MRI) (Figure 4) [6,11].

On the other hand, positron-emission tomography (PET) is reserved for staging and the detection of extrathoracic disease. The radiologist should always evaluate the location and extent of the tumor, as well as specifically describe any infiltration to the heart and major vessels, along with complications, such as superior vena cava obstruction [11]. For male patients, a testicular ultrasound should always be considered in patients with germ cell tumors, even if the primary location appears to be in the mediastinum.

Alpha-fetoprotein (AFP) is a specific marker for YST, but it lacks sensitivity since elevated levels can also be observed in other conditions, such as hepatocellular carcinoma. However, in cases where a mediastinal mass has been detected, the finding of increased AFP levels makes the diagnosis of an endodermal sinus tumor highly probable. This germ-cell variant is almost always accompanied by significantly elevated AFP levels (>1000 ng/mL), aiding in diagnosis. This marker is also useful for postoperative monitoring of treatment success. It is also important to differentiate primary mediastinal YST from other mediastinal germ cell tumors, such as seminomas and embryonal carcinomas, for which β-human chorionic gonadotropin is specific (β-hCG). Mediastinal embryonal carcinomas often exhibit positive β-hCG reactivity, whereas YSTs typically do not [15].

Preoperative percutaneous needle biopsy is generally not mandatory [4,11,16]. In cases of mixed germ cell tumors with minor components of YST, biopsy analysis may fail to detect the YST component due to its typically limited presence in small malignant foci within the larger neoplasm [17].

## 4. Treatment Modalities

Tumors exhibit variable biological behavior and prognosis, according to subtype. For example, seminomas respond well to cisplatin and teratomas have an excellent prognosis after complete resection, while other subtypes have a long-term survival rate of 54% after multimodality treatment [4,18]. In the past, the prognosis was a life expectancy of less than 6 months. However, this prognosis has been considerably improved by the breakthrough of platinum chemotherapy combined with consolidation surgery. Contemporary treatment protocols include four cycles of bleomycin, etoposide, and cisplatin (BEP) or ifosfamide, etoposide, and cisplatin (VIP), followed by surgical resection of residual masses, which enable long-term survival in approximately 40% of patients [4,5,9,10,11]. This approach has significantly improved outcomes for patients with YST, offering a better prognosis compared to earlier therapeutic strategies. Two additional cycles of chemotherapy may be considered if viable malignancy is detected after surgery [11]. In cases of end-stage renal insufficiency, drugs exhibit limited effectiveness, and thus, radical surgery may be considered instead of chemotherapy. Notably, bleomycin and ifosfamide are not effectively cleared by hemodialysis, leading to increased toxicity, including pulmonary fibrosis or seizures [19]. Alternative treatments may include other medications, like cisplatin and etoposide, at reduced doses [20]. Before starting treatment, an anatomical assessment of the lung using radiological imaging, such as a CT scan, should always be performed to determine whether a potential lung resection may be required. Chemotherapy with VIP is preferred over BEP to minimize pulmonary complications in patients who often require extensive surgical resection, due to the risk of lung toxicity associated with bleomycin. The patient’s overall clinical status, including pulmonary (such as diffusion capacity-DLCO) and hematological function, should be evaluated both before and after chemotherapy to aid in surgical planning and intraoperative decision-making [21]. Following the completion of chemotherapy, the administration of granulocyte colony-stimulating factor (GCSF) may be necessary [9]. Moreover, radiotherapy may be an effective therapeutic option in cases of postoperative recurrence of a chemotherapy-resistant tumor [22].

To the best of our knowledge, no targeted therapy or immunotherapy have been described for the treatment of mediastinal YST to date, although attempts have been made to treat other extragonadal types. In the study by Wang et al., a combination of bevacizumab and tislelizumab was used as an adjunct to chemotherapy, based on the current comprehensive treatment approach for epithelial ovarian cancer [23]. This approach is optimal for improving the survival of patients with advanced recurrent non-endometrial YST; however, it specifically targets the location of this particular tumor and its metastases. Despite the very limited clinical activity of immunotherapy in patients with germ cell tumors, some published data exist regarding the use of anti-PD-1 agents, such as pembrolizumab, and studies utilizing nivolumab in patients with high PD-L1 expression [24,25]. Nevertheless, these findings do not unequivocally demonstrate a favorable response in patients, because of the complexity of the mechanism and heterogeneity of the tumor immune environment. Immunotherapy targeting Glypican-3 (GPC3) represents the next promising path for cancer treatment, including yolk sac tumors. However, further studies are required to validate the efficacy of this approach, both in YST and in other tumor types [26,27].

**Figure 5:**
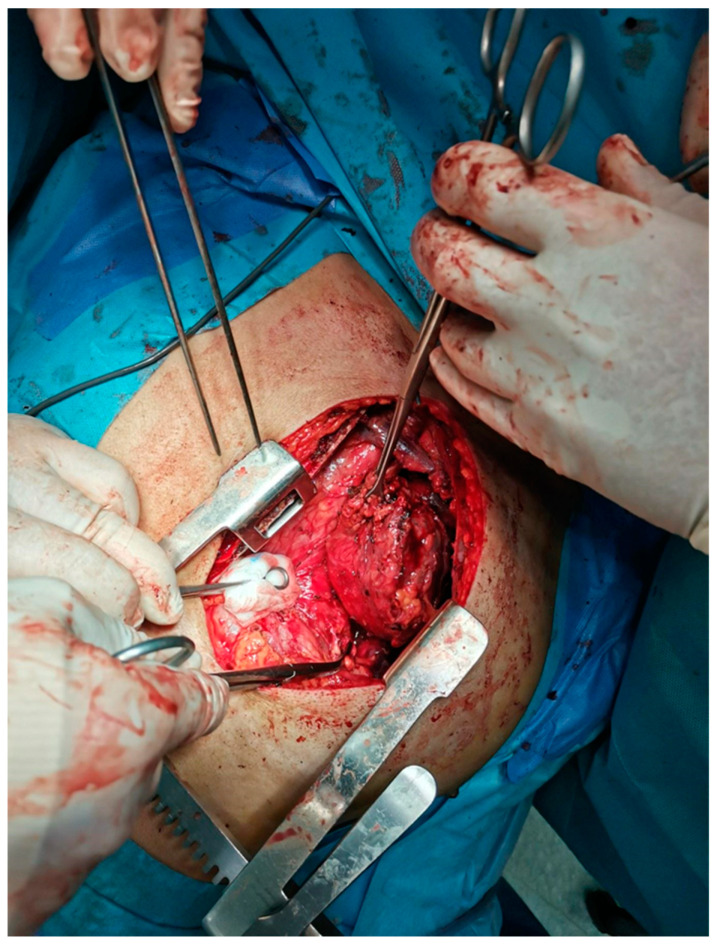
Resection of YST located in the anterior mediastinum.

The rapid expansion of YST and extensive infiltration of adjacent structures significantly raises the complexity of surgical interventions, which may require cooperation among different surgical specialties (e.g., for initiating cardiac circulatory bypass). Additionally, the lack of published data regarding optimal surgical planning and potential approaches means that the surgical team must tailor a highly individualized approach for each patient [6]. The choice of surgical treatment for YST mainly depends on the location. In our review, we focused on resection techniques for mediastinal tumors, as this is the most common location of extragonadal germ cell tumors [28]. The high effectiveness of surgical interventions is confirmed by the high percentage of patients with negative surgical margins of resection for the group of 56 patients with primary mediastinal germ cell tumors (PMGCT). Only two cases had positive surgical resection margins, including one with YST who received neoadjuvant chemotherapy [29]. Furthermore, it is important to highlight that surgery is a treatment strategy for PMGCT involving resection of residual disease with a tumor histology other than a seminoma [30]. Patients with endodermal sinus tumors are most often adolescents and exhibit the highest rate of cancer-specific survival among cases of primary mediastinal non-seminomas. Among patients in this group with YST, 43.2% were treated with radical surgery or total resection [6]. These tumors often cause substantial compression-related symptoms, requiring an approach that reduces the tumor size [31]. Confirmation is provided by a treatment using three cycles of neoadjuvant VIP chemotherapy, surgical treatment, and one dose of adjuvant VIP chemotherapy in an 18-year-old man with YST. The patient had no meta and a pathological response was achieved at the five-month follow-up [32]. The resection of YST is particularly challenging because these tumors develop wide and strong adhesion to proximal large vessels and lungs. YST sometimes exhibits a cystic component as shown in Figure 5.

Additionally, the potential contamination of the field with malignant cells is always an important consideration. The preferred technique of choice for small tumors located in the anterior mediastinum is sternotomy. For large tumors of the anterior mediastinum, the combination of upper median sternotomy and anterior thoracotomy is a useful strategy. Furthermore, the use of the hemi-clamshell and its variants in such cases gives a satisfactory result. When a tumor involving both breasts is operated on, a clamshell incision is performed. If access to the mediastinal vascular structures is desired, a lateral thoracotomy is performed. In the case of residual tumors, it is worth considering separating the mass from the adjacent structures with en bloc resection. This is possible when only fibrosis is visible [21]. Furthermore, the method of resection of the residual tumor was presented for a neuroendocrine tumor located in the anterosuperior mediastinum. Excision of the mass was performed via sternotomy into the left fourth intercostal space. Subsequently, the left anonymous vein was noted in the surgical field near the tumor. In addition, the apical segments of the lung and pericardium on the left side were infiltrated. The first step was to identify the vagus and recurrent nerve, followed by pulmonary wedge resection. After careful assessment of the superior vena cava and left innominate vein, the tumor mass was removed with part of the pericardium. Using a bovine patch, pericardial reconstruction was performed [33]. Chaudry et al. provide the best description of resecting a large cystic endodermal sinus tumor [9]. In their described case, median sternotomy and tumor localization were followed by the placement of a purse-string suture in the soft area of the tumor, with subsequent placement of a clamped 32F drain and tightening of the purse-string suture around the drain. After the contents of the mass were evacuated, the tumor was detached from the great vessels, along with part of the pericardium.

It is unusual for YST to be located in the posterior mediastinum. Tanase et al. described a case in which the patient was diagnosed with an extramucosal esophageal tumor, which was an endodermal sinus tumor [34]. On this patient, they performed a McKeown procedure typical for esophageal cancer, using a right thoracotomy. No lesion was detected in the pleural cavity, only in the posterior mediastinum. They additionally performed a wedge resection of the right lower lung lobe. The surgical team carefully released the esophagus from surrounding structures. The esophagus was stapled and cut at the apex of the pleural cavity, followed by right pleural cavity drainage and thoracic wound closure. Next, the patient was repositioned, and a laparotomy was conducted, revealing no metastatic lesions. Moreover, a celiac lymphadenectomy was performed on tissues invaded by the tumor. After the removal of the esophagus and part of the stomach, an Akiyama gastric tube was prepared and pulled through the posterior mediastinum. Next, the team performed a modified Collard esophagogastric anastomosis and completed the procedure with a jejunostomy and drainage. This two-stage procedure involving both thoracotomy and laparotomy requires optimal preoperative preparation for patients who are fit for surgery.

Another particularly challenging approach has been described for primary thoracic dumbbell YST, presenting with both epidural and extraspinal extension and severe spinal cord compression at different thoracic levels [35]. In this case, surgical intervention was the first treatment step due to a symptomatic progressive tumor at the thoracic vertebral level. The surgery began with the removal of the extraspinal component, followed by the removal of the left T8 vertebral pedicle, zygapophyseal joint, and part of the 8th left rib. Next, the extraspinal component was exposed and thoroughly resected. In the final stage, spinal cord stabilization systems were applied.

The role of minimally invasive surgery in the treatment of mediastinal YST is not well-documented. Due to the large size, aggressive nature, and strong adhesions of these tumors to the surrounding structures, open approaches, such as sternotomy or thoracotomy, are often required to perform the extensive resections needed for complete tumor removal. To our knowledge, minimally invasive techniques, such as laparoscopy, have only been documented in non-mediastinal cases, specifically in patients with more traditionally located abdominal tumors [36,37].

## 5. Discussion

Germ cell tumors of the mediastinum are invasive, requiring multimodal treatment, often including extensive resection by sternotomy or thoracotomy. Despite the intricate nature of these procedures, operative details have not been adequately described or analyzed in the literature [18]. Some studies recognize that mediastinal YST resections are complex operations requiring high skill and experience [10,38]. To achieve long-term survival, the first treatment option is generally not surgical treatment, but rather neoadjuvant chemotherapy with subsequent surgical removal of the residual mass [5]. Huang et al. reported that performing resection in the second phase of treatment yields a higher resection rate and is more beneficial than surgery at the beginning of treatment [39].

Therefore, optimal timing for tumor resection is very important. Surgery is generally planned based on AFP levels and the reduction in tumor size. Once a good response is achieved, surgery is typically scheduled to begin around 4 weeks after the completion of chemotherapy to allow for bone marrow recovery. However, the lack of a decrease in serum AFP does not contraindicate the initiation of surgery. Complete normalization of serum AFP levels occurs in fewer than 5% of patients but is an important prognostic factor [21,40,41,42]. Dabsha et al. conducted a meta-analysis of case reports comparing various factors, including treatment approaches and the stage of mediastinal YST [43]. Their findings indicated that the use of surgery, chemotherapy, and tumor location in the anterior mediastinum was associated with a clear trend toward improved survival. However, unlike other studies reporting 5-year survival rates of 50–60%, they observed a 5-year survival rate of 22.9% in the adult group [21,38,43,44]. This discrepancy is likely due to the rarity of the disease and the fact that other studies often encompass the broader category of PMGCT, rather than focusing solely on YST. Notably, initial surgical intervention can be considered in some cases with co-existent active medical problems, such as end-stage renal failure [19]. Tanaka’s group has demonstrated the individual tailoring of treatment strategy based on case characteristics [45]. Their first-described case involved a tumor measuring 14.4 × 9.8 × 10.0 cm in the anterior mediastinum, causing compression of the heart. They administered five initial cycles of chemotherapy and obtained satisfactory tumor resection, with a follow-up of 32.5 months. Their second case had a mass with a similar location but with significantly smaller dimensions of 7.6 × 6.5 × 3.5 cm. This case was treated with surgical removal, followed by four successive cycles of chemotherapy, which led to a satisfactory 3-year follow-up. Notably, the first case exhibited laboratory AFP levels of 31,536 ng/mL and normal hCG, while the second case showed an AFP level of 3034 ng/mL and hCG level of 20.6 ng/mL. Based on these cases, we can conclude that the choice of treatment strategy depends on both the mass location and size, as well as the AFP and hCG levels.

Kesler et al. identified three factors that influence long-term survival—AFP levels after chemotherapy, the pathological status of the residual mass, and the presence of pulmonary metastasis [38]. In 1999, Kesler et al. reviewed 40 cases of primary non-seminomatous tumors, which showed a 48-month overall survival rate of 61%. They concluded that resections after chemotherapy were severely complicated by the extensive mediastinal fibrosis and the potential need for a cardiopulmonary bypass [38]. Although the use of cardiopulmonary bypass for large mediastinal masses is a well-established technique, it must be noted that, in addition to their large size, YST also causes significant fibrosis that complicates the cannulation process and tissue dissection. This aspect of complexity has not been thoroughly discussed. Kessler performed cardiopulmonary bypass twice in cases of non-seminomatous tumors and May M. applied it in a case of intra-cardiac metastasectomy [46]. In these situations, cardiac cannulation is technically more challenging and can even be risky, such that peripheral cannulation may be the only feasible option. Surgeons should master peripheral cannulation techniques since non-ideal cannulation conditions are expected.

In another study, Li Qin et al. demonstrated the extent and complexity of the surgical management of these tumors [10]. They reported the resection of 15 YST with R0 margins achieved in 11 patients and R2 margins achieved in the remaining 4 patients. In 40% of cases, the patients underwent partial pericardiectomy in addition to pulmonary wedge resection with mediastinal lymphadenectomy. This study achieved a progression-free survival rate (PFSR) of 66.7% at 1 year and 60% at 2 years, with an estimated 2-year overall survival rate of 73.3%. All patients experienced disease recurrence within 6–11 months of resection (median of 8.0 months), highlighting the need for close follow-up. This need for follow-up has also been indicated by Koizumi et al. [16]. Their publication shared insights from a 30-year period during which they treated 22 mediastinal germ cell neoplasms, including three cases of YST. They demonstrated that long-term survival and even cure in some cases could be achieved with adequate chemotherapy followed by local surgical treatment. Their study highlighted the development of secondary malignancies, which necessitates a close and long-term follow-up of these patients. However, despite their wealth of experience, Koizumi et al. reported no details concerning the resections. Similarly, no surgical details were included in the well-known study by MD Anderson Cancer Center [4]. They recommended the use of neoadjuvant chemotherapy followed by resection surgery, based on conclusions drawn from 27 cases of mediastinal masses, ranging from 3 to 20 cm in diameter, with metastasis present in 65% of patients. In a 2018 report, Liu et al. presented seven cases of YST [11]. Surgical management included extensive resections, requiring extended resection of the tumors, along with resection of adjacent structures (e.g., partial pericardiotomy, superior vena cava, phrenic nerve, and pulmonary wedge resections, as well as the extended thymectomy discussed earlier in this review). They achieved R0 resection margins in all cases but one and their preferred operative approaches were muscle-sparing thoracotomy and sternotomy in cases involving the lung or great vessels.

## 6. Conclusions

Clinicians facing the multimodality treatment of mediastinal yolk sac tumors and aiming to perform consolidation surgery should appreciate the complexity and risks of the operation. It is mandatory to perform a full preoperative work-up for cardiothoracic patients. Moreover, patients should be informed about the procedure’s complexity, perioperative risks, and outcomes. Resections may range from esophageal and lung resections to cardiovascular on-pump procedures. To achieve satisfactory outcomes, resections must be performed at high-volume centers with robust surgical experience and extracorporeal support facilities.

## Figures and Tables

**Table 1:** Eligibility criteria according to the PICOTS framework.

	Inclusion Criteria	Exclusion Criteria
Patient	Patients with confirmed diagnosis of yolk-sac tumor	-
Intervention	Diagnostics, pharmacological or surgical treatment	-
Comparator	Control case or cases not required	-
Outcomes	Final diagnosis, successful treatment	-
Timeframe	Papers published since 1949	-
Study design	Case report or case series	Articles in languages other than English
